# BRAF V600E Mutations Are Common in Pleomorphic Xanthoastrocytoma: Diagnostic and Therapeutic Implications

**DOI:** 10.1371/journal.pone.0017948

**Published:** 2011-03-29

**Authors:** Dora Dias-Santagata, Quynh Lam, Kathy Vernovsky, Natalie Vena, Jochen K. Lennerz, Darrell R. Borger, Tracy T. Batchelor, Keith L. Ligon, A. John Iafrate, Azra H. Ligon, David N. Louis, Sandro Santagata

**Affiliations:** 1 Department of Pathology and Center for Cancer Research, Massachusetts General Hospital and Harvard Medical School, Boston, Massachusetts, United States of America; 2 Division of Hematology-Oncology, Massachusetts General Hospital Cancer Center and Harvard Medical School, Boston, Massachusetts, United States of America; 3 Department of Medical Oncology, Dana–Farber Cancer Institute, Boston, Massachusetts, United States of America; 4 Center for Molecular Oncologic Pathology, Dana–Farber Cancer Institute, Boston, Massachusetts, United States of America; 5 Department of Neurology and Department of Radiation Oncology, Massachusetts General Hospital, Boston, Massachusetts, United States of America; 6 Department of Pathology, Children's Hospital Boston and Harvard Medical School, Boston, Massachusetts, United States of America; 7 Department of Pathology, Brigham and Women's Hospital and Harvard Medical School, Boston, Massachusetts, United States of America; Stanford University School of Medicine, United States of America

## Abstract

Pleomorphic xanthoastrocytoma (PXA) is low-grade glial neoplasm principally affecting children and young adults. Approximately 40% of PXA are reported to recur within 10 years of primary resection. Upon recurrence, patients receive radiation therapy and conventional chemotherapeutics designed for high-grade gliomas. Genetic changes that can be targeted by selective therapeutics have not been extensively evaluated in PXA and ancillary diagnostic tests to help discriminate PXA from other pleomorphic and often more aggressive astrocytic malignancies are limited. In this study, we apply the SNaPshot multiplexed targeted sequencing platform in the analysis of brain tumors to interrogate 60 genetic loci that are frequently mutated in 15 cancer genes. In our analysis we detect BRAF V600E mutations in 12 of 20 (60%) WHO grade II PXA, in 1 of 6 (17%) PXA with anaplasia and in 1 glioblastoma arising in a PXA. Phospho-ERK was detected in all tumors independent of the BRAF mutation status. BRAF duplication was not detected in any of the PXA cases. BRAF V600E mutations were identified in only 2 of 71 (2.8%) glioblastoma (GBM) analyzed, including 1 of 9 (11.1%) giant cell GBM (gcGBM). The finding that BRAF V600E mutations are common in the majority of PXA has important therapeutic implications and may help in differentiating less aggressive PXAs from lethal gcGBMs and GBMs.

## Introduction

Pleomorphic xanthoastrocytoma (PXA) is an uncommon low-grade glial neoplasm of the central nervous system that most frequently affects children and young adults [1]–[6]. The tumors often arise in the temporal lobe and involve both the superficial cortex and the overlying meninges [7]–[9]. During the clinical evaluation of PXA resection specimens, the dense cellularity and pleomorphism typical of PXA raises a differential diagnosis including ganglioglioma and a range of high-grade malignancies like glioblastoma (GBM), giant cell glioblastoma (gcGBM), gliosarcoma or pleomorphic sarcoma [1], [5]. The presence of eosinophilic granular bodies and the compact, predominantly non-infiltrating nature of PXA also can introduce pilocytic astrocytoma into the differential diagnosis [5]. The histopathologic diagnosis of PXA can often be accomplished readily when the histologic findings are carefully considered in light of the clinical and radiological features, although the diagnosis can be challenging and confusion with other neoplasms can occur [10]. A reproducible ancillary diagnostic marker for PXA would, therefore, be of significant clinical use.

PXA are typically slow growing at presentation and as a result patients commonly experience long-standing seizures. While the prognosis is relatively favorable, the recurrence rate following resection is 30% within five years and about 40% within ten years. The overall survival rate is 80% at five years and 70% at ten years [9], [11]. In its classic form, the histology of the tumors is distinct with large pleomorphic giant cells demonstrating xanthomatous change, a dense deposition of intercellular reticulin and the presence of eosinophilic granular bodies [12]. Anaplastic changes such as high mitotic activity and necrosis are uncommon at initial presentation but become increasingly frequent in recurrences [7], [13]. The treatment of PXA typically involves surgical resection followed by radiological monitoring [13]. Recurrent lesions or tumors that demonstrate anaplastic features at primary resection are treated with radiation [14] and chemotherapeutic protocols [7], [10] that are also used for anaplastic astrocytoma and glioblastoma. The development of more selective targeted therapies for PXA and the design of future clinical trials are dependent on an understanding of the molecular genetic lesions that drive its pathogenesis.

Identification of genetic lesions in PXA has been challenging for a number of reasons. Most notably, the number of patients with PXA is low even in large academic hospital centers resulting in a paucity of cases for investigation. Since few tumor banks have frozen PXA samples most analyses must be done on formalin-fixed paraffin-embedded (PXA) tissue and robust technologies for analyzing DNA from FFPE sources have only recently emerged [15]–[17]. In addition, PXA cell lines are not available and animal models have not been generated. To date, molecular genetic analysis of PXA has been predominantly limited to *TP53* revealing mutations in 6% of cases (7 of 123) [18]–[21]. There is no correlation between mutated *TP53* and the presence of anaplastic features. While amplifications of the *EGFR*, *MDM2* or *CDK4* loci are absent [18], array CGH analysis demonstrated homozygous loss of the 9p21.3 locus containing *CDKN2A/CDKN2B* in 6 of 10 cases [22].

Recently, multiplexed targeted sequencing platforms have been developed that can be used to simultaneously investigate the mutation status of multiple key cancer genes [15]–[17]. These assays are capable of robustly identifying mutations in DNA derived from FFPE samples and allow for the rapid identification of clinically important and therapeutically tractable genetic mutations. In this study, we use the SNaPshot mutation profiling assay to investigate the mutation status of 15 cancer associated genes in 26 PXA and 71 GBM.

## Materials and Methods

### Tissue Samples and Pathology Review

Paraffin blocks from surgical resection specimens covering a 14-year period (1996–2010) were obtained from the archives of Massachusetts General Hospital (MGH), Brigham and Women's Hospital (BWH) and Children's Hospital Boston (CH) in accordance with the regulations for excess tissue use stipulated by the review boards of each institution. Cases were retrieved for pathology review if the clinical diagnosis was gcGBM, PXA or if the tumor was thought to display features suggestive of PXA. Hematoxylin and eosin (H&E)-stained sections of all cases were reviewed for the presence of pleomorphic cells, xanthomatous cells, reticulin, eosinophilic granular bodies, inflammation, and calcifications, necrosis and microvascular proliferation. World Health Organization diagnostic criteria were used for assigning histopathology diagnoses [2]. Six of the PXA II samples in this study are also included in a separate study by Schindler et al [23].

### Ethics Statement

This study was conducted according to the principles outlined in the Declaration of Helsinki. All work on human tissues was conducted on excess archival human material from the Departments of Pathology at MGH, BWH and CH. The research study was approved by the Institutional Review Board for Human Research at MGH, BWH and CH as an excess tissue protocol and the ethics committee specifically waived the need for consent. The study was designed in light of recommendations from the STARD (STAndards for the Reporting of Diagnostic accuracy studies) statement. http://www.stard-statement.org/.

### SNaPshot Sample Preparation

H&E-stained slides derived from representative surgical specimens were evaluated for the presence of tumor. Available tumor tissue was manually macro-dissected from serial 4-micron unstained sections, or cored from the paraffin block using a 1.5-mm dermal punch. Total nucleic acid was extracted from FFPE tumor tissue using the FormaPure Kit (Agencourt Bioscience Corporation, Beverly, MA) on a Beckman Coulter Biomek NX^P^ workstation.

### SNaPshot Genotyping

Our group has recently developed a tumor genotyping assay to test tumor specimens prospectively and to guide clinical decision making toward targeted therapies [15]. This clinical assay is based on the SNaPshot method (Applied Biosystems) and consists of multiplexed PCR and multiplexed single-base extension, followed by capillary electrophoresis. The original tumor profiling panel described by Dias-Santagata *et al.* [15], was expanded to include four additional assays that test for hotspot mutations in the isocitrate dehydrogenase genes 1 (*IDH1*) and 2 (*IDH2*), recently shown to be frequently altered in gliomas [24]. The genotyping analysis queried 60 commonly mutated loci in 15 cancer genes (Table 1) testing for 140 previously described somatic mutations (COSMIC database, v49 release). SNaPshot genotyping was performed using previously described conditions [15]. Briefly, genetic regions flanking the loci of interest were co-amplified using eight multiplexed PCR primer pools and 60 ng of total nucleic acid. PCR amplification (95°C×8 min; 45 cycles of 95°C×20 s, 58°C×30 s, 72°C×1 min) was followed by treatment with shrimp alkaline phosphatase and exonuclease I (USB, Cleveland, OH). Specific mutation loci were tested using 8 pools of extension primers, each annealing immediately adjacent to the nucleotide position of interest. A multiplexed single-base extension reaction that adds a fluorescently labeled ddNTP to each locus-specific probe was performed, using the ABI SNaPshot Multiplex Ready Reaction Mix (96°C×30 s; 25 cycles of 96°C×10 s, 50°C×5 s and 60°C×30 s). Labeled extension products were resolved by capillary electrophoresis on an automatic DNA sequencer (ABI PRISM 3730 DNA Analyzer, Applied Biosystems) and data analysis was performed using GeneMapper Analysis Software version 4 (Applied Biosystems).

**Table 1:** SNaPshot tumor genotyping panel.

Gene	Amino acid - cDNA residue	Gene	Amino acid - cDNA residue
*APC*	R1114 - 3340C	*KRAS*	G12 - 34G
*APC*	Q1338 - 4012C	*KRAS*	G12 - 35G
*APC*	R1450 - 4348C	*KRAS*	G13 - 37G
*APC*	T1556fs* - 4666_4667insA	*KRAS*	G13 - 38G
*BRAF*	V600 - 1798G	*NOTCH1*	L1575 - 4724T
*BRAF*	V600 - 1799T	*NOTCH1*	L1601 - 4802T
*CTNNB1*	D32 - 94G	*NRAS*	G12 - 34G
*CTNNB1*	D32 - 95A	*NRAS*	G12 - 35G
*CTNNB1*	S33 - 98C	*NRAS*	G13 - 37G
*CTNNB1*	G34 - 101G	*NRAS*	G13 - 38G
*CTNNB1*	S37 - 109T	*NRAS*	Q61 - 181C
*CTNNB1*	S37 - 110C	*NRAS*	Q61 - 182A
*CTNNB1*	T41 - 121A	*NRAS*	Q61 - 183A
*CTNNB1*	T41 - 122C	*PIK3CA*	R88 - 263G
*CTNNB1*	S45 - 133T	*PIK3CA*	E542 - 1624G
*CTNNB1*	S45 - 134C	*PIK3CA*	E545 - 1633G
*EGFR*	G719 - 2155G	*PIK3CA*	Q546 - 1636C
*EGFR*	T790 - 2369C	*PIK3CA*	Q546 - 1637A
*EGFR*	L858 - 2573T	*PIK3CA*	H1047 - 3139C
*EGFR*	E746_A750 - 2235_2249del	*PIK3CA*	H1047 - 3140A
*EGFR*	E746_A750 - 2236_2250del	*PIK3CA*	G1049 - 3145G
*FLT3*	D835 - 2503G	*PTEN*	R130 - 388C
*IDH1*	R132 - 394C	*PTEN*	R173 - 517C
*IDH1*	R132 - 395G	*PTEN*	R233 - 697C
*IDH2*	R172- 514A	*PTEN*	K267fs*- 800delA
*IDH2*	R172 - 515G	*TP53*	R175 - 524G
*JAK2*	V617 - 1849G	*TP53*	G245 - 733G
*KIT*	D816 - 2447A	*TP53*	R248 - 742C
		*TP53*	R248 - 743G
		*TP53*	R273 - 817C
		*TP53*	R273 - 818G
		*TP53*	R306 - 916C

The panel tests 60 frequently mutated loci in 15 cancer genes. Because several nucleotide variants have been described at each site, this assay can detect 140 previously described mutations according to the COSMIC database, v49 release (http://www.sanger.ac.uk/genetics/CGP/cosmic/).

The following primers were used for targeted mutation analysis at codon R132 in *IDH1* (nucleotide positions c.394 and c.395) and at the equivalent amino acid R172 in *IDH2* (nucleotide positions c.514 and c.515): (PCR primers: *IDH1* exon 4, 5′-ACGTTGGATGGGCTTGTGAGTGGATGGGTA-3′ (forward) and 5′- ACGTTGGATGGCAAAATCACATTATTGCCAAC-3′ (reverse) and *IDH2* exon 4, 5′- ACGTTGGATGAACATCCCACGCCTAGTCC-3′ (forward), and 5′- ACGTTGGATGCAGTGGATCCCCTCTCCAC-3′ (reverse). Extension primers: IDH1.394 extR 5′-GACTGACTGGACTGACTGACTGACTGACTGGACTGACTGACTGAGATCCCCATAAGCATGAC-3′, IDH1.395 extR 5′-TGATCCCCATAAGCATGA-3′, IDH2.514 extF 5′-GACTGACTGACTGACTGACTGACTGACTGGACTGACTGACTGACTGACTGGACTGACTGACCCATCACCATTGGC-3′ and IDH2.515 extR 5′- GACTGACTGACTGACTGACTGACTGACTGACTGACTGGACTGACTGACTGACTGACTGGACTGACTGAGCCATGGGCGTGC-3′). The primer sequences for the other assays were previously described [15].

### Slide Preparation, Immunohistochemistry, Histology Staining and Scoring

Clinical specimens that had been fixed in 10% buffered-formalin and prepared as FFPE tissue blocks were used to generate four-micron sections. The slides were stained with H&E. Serial sections of the paraffin blocks were cut and these slides were used for immunohistochemistry (IHC) as well as mutation analysis. For IHC, tissue sections were deparaffinized and rehydrated through xylene/graded alcohols. Endogenous peroxidase activity was blocked with 3% hydrogen peroxide (15 min incubation) in 100% alcohol (1∶1). Heat-induced antigen retrieval was carried out with Dako Target Retrieval Solution, pH 6.0, and pressure cooker heated (120+/−2°C) for 30 seconds at 15+/−5 psi. Sections were stained for phospho-p44/42 MAPK (Erk1/2) (Thr202/Tyr204) using a 1∶500 dilution of rabbit monoclonal antibody (#4370: Phospho-p44/42 MAPK (Erk1/2) (Thr202/Tyr204) (D13.14.4E) from Cell Signaling Technology (Beverly, MA). The antibody was optimized using SignalSlide™ Phospho-p44/42 MAPK (Thr202/Tyr204) IHC Controls from Cell Signaling (#8103) that consist of paraffin-embedded NIH/3T3 cells that are either untreated or PMA-treated. The antibody is reported to detect endogenous levels of p44 and p42 MAP Kinase (Erk1 and Erk2) when phosphorylated at residues Thr202 and Tyr204 of Erk1 and (Thr185 and Tyr187 of Erk2), and singly phosphorylated at Thr202. The antibody is reported not to cross-react with the corresponding phosphorylated residues of either p38 MAP or JNK/SAPK kinases. Application of the primary antibodies was followed by a 30-minute incubation with Dako Labeled Polymer-HRP anti-rabbit IgG secondary antibody, and visualized with 3, 3′– diaminobenzidine (DAB) as a chromogen (Envision+ System) with Mayer hematoxylin counterstaining. Grading of phospho-p44/42 MAPK immunoreactivity was based on the following semiquantitative approach: 0, no tumor cells demonstrating staining; 1+, >0–25% of tumor cells reactive; 2+, >25%–50% of tumor cells reactive; 3+, >50%–75% of tumor cells reactive; 4+, >75% of cells reactive. Giant cell glioblastoma sections were stained with a monoclonal antibody directed against p53 (clone DO-1, Immunotech, Marseille, France) at a 1∶1200 dilution using similar conditions as described above for the phospho-p44/42 MAPK (Erk1/2). Reticulin staining was performed with Reticulin/Nuclear Fast Red Stain Kit, Artisan™ (DAKO, Carpinteria, CA), according to the manufacturer's recommendations.

### Fluorescence In Situ Hybridization

Fluorescence *in situ* hybridization (FISH) was performed on four micron tissue sections using the D7Z1 DNA Probe (chromosome 7 alpha satellite DNA) (Abbott Molecular) at 7p11.1-q11.1 and homebrew probes (purchased from CHORI; www.chori.org) RP11-767F15 and RP11-60F17 that map to 7q34. The RP11-767F15 probe includes the 5′ region of *BRAF* and the RP11-60F17 probe includes the 3′ region of *BRAF*. *BRAF* duplication status was assessed in 50 tumor nuclei per case.

### Statistical Analysis

Statistical Analysis consisted of Fisher's exact test (association of dichotomous factors), or t-test (comparison of means). Data analysis was conducted using Prism 5.0b (GraphPad Software Inc., San Diego, CA) and significance was defined as P<0.05.

## Results

### Glioma Cases Profiled with SNaPshot

**Figure 1:**
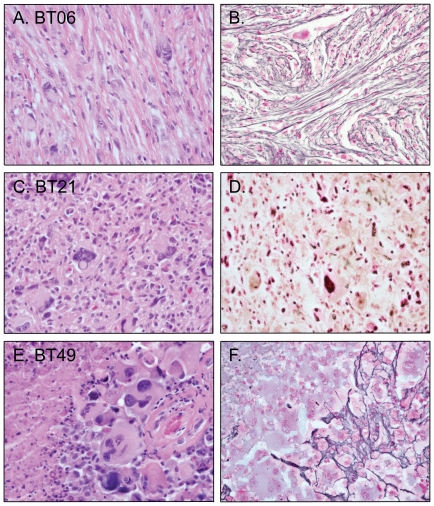
Representative photomicrographs of tumors. A. H&E-stained section of a pleomorphic xanthoastrocytoma (PXA, BT06) demonstrating fascicular growth pattern and prominent intercellular reticulin deposition (B) corresponding to PXA (m). C. H&E-stained section of PXA (BT21) that demonstrates neither a fascicular growth pattern nor prominent intercellular reticulin deposition (D). E. H&E-stained section of a giant cell glioblastoma (gcGBM) arising from a PXA (BT49) with marked pleomorphism and giant cells and multifocal reticulin deposition (F).

To investigate the genetic changes underlying pleomorphic xanthoastrocytomas, we identified relevant tumors that were resected between 1996 and 2010 at Massachusetts General Hospital, Brigham and Women's Hospital, and Children's Hospital Boston. We retrieved cases that were thought to be unequivocal grade II PXA during the clinical evaluation of the surgical pathology specimens as well as tumors that demonstrated most of the features of PXA and were thought to be “consistent with PXA.” In addition, we identified cases of PXA with anaplastic features (aPXA) such as necrosis and increased mitoses as well as cases of giant cell glioblastoma (gcGBM, grade IV). Representative images are illustrated in Figure 1. In all, we retrieved 36 resection specimens of astrocytomas with marked cellular pleomorphism from 35 patients, with one patient having both a primary (BT29) PXA resected and then a recurrence of a PXA with anaplastic features (BT 41). Thirty of the cases were primary tumors and six were recurrences. Five of the six recurrences had primary resections performed at an outside hospital and the primary surgical specimen was not available for genotyping. In total, we analyzed 20 cases of grade II PXA and six cases of aPXA, (Table 2). We also analyzed nine gcGBM since this variant of glioblastoma is commonly included in the differential diagnosis of PXA. The age distribution of patients with grade II PXAs was 7 years to 71.5 years with mean and median ages at presentation of 25.8 years and 19.1 years, respectively. The age distribution of patients at the time of aPXA resection was 29.3 years to 76.2 years with mean and median ages at presentation of 57.4 years and 54.3 years, respectively. The grade II PXAs histologically fell into two general types: those that had predominantly a distinct mesenchymal-like growth pattern with fascicular and sometimes storiform growth pattern (PXA (m)) (Fig. 1A and B) and those that predominantly had a diffuse fibrillary background (Fig. 1C and D). PXA (m) tumors were more likely to demonstrate intercellular reticulin deposition. Interestingly, all nine of the PXAs with prominent mesenchymal-like growth originated in the temporal lobe. In contrast, only five of the remaining 11 PXA cases (45%) were found in the temporal lobe, with others located in frontal lobe (n = 2), suprasellar region (n = 1), and in the parietal/occipital lobes (n = 3). The mean age for grade II PXA with prominent mesenchymal-like growth was 25.8 years, whereas the remaining grade II PXAs had a mean age of 25.7 years.

**Table 2:** Clinical Data and Summary of Results.

Case #	Hospital	Gender	Age (yrs)	Size (cm)	Side	Location	Histology Classification	SNaPshot Result	BRAF FISH	pERK	Reticulin	Specimen Genotyped
BT06	CH	F	7.0	3.4	Right	Temporal	PXA (m)	BRAF V600E(c.1799T>A)	No duplication	1	Present	Recurrence
BT07	CH	F	10.9	2.2	Left	Temporal	PXA	No mutations	No duplication	3	Absent	Primary
BT19	MGH	M	15.3	1	Right	Temporal	PXA (m)	No mutations	NA	4	Present	Primary
BT20	MGH	M	42.8	0.8	Left	Occipital	PXA*	BRAF V600E(c.1799T>A)	NA	4	Present	Primary
BT21	MGH	M	19.7	0.9	Left	Temporal	PXA	BRAF V600E(c.1799T>A)	No duplication	3	Focal	Primary
BT22	MGH	M	43.5	6.8	Left	Frontal	aPXA	BRAF V600E(c.1799T>A)	No duplication	4	Focal	Recurrence
BT23	MGH	F	47.1	1.2	Right	Suprasellar	PXA	No mutations	No duplication	2	Present	Primary
BT24	MGH	F	12.0	2.6	Right	Temporal	PXA (m)	BRAF V600E(c.1799T>A)	NA	4	Present	Primary
BT25	BWH	F	10.2	1.5	Left	Frontal	PXA	No mutations	No duplication	4	Present	Primary
BT26	BWH	M	71.5	2	Right	Temporal	PXA (m)	BRAF V600E(c.1799T>A)	No duplication	2	Present	Primary
BT27	BWH	M	37.9	6	Right	Temporal	PXA (m)	BRAF V600E(c.1799T>A)	No duplication	1	Present	Primary
BT28	BWH	M	18.8	NA	Right	Parietal	PXA	No mutations	No duplication	2	Focal	Recurrence
BT29	BWH	M	45.7	0.6	Right	Temporal	PXA	No mutations	No duplication	4	Focal	Primary
BT30	BWH	M	29.3	3	Right	Frontal	aPXA	No mutations	No duplication	4	Present	Primary
BT31	BWH	M	29.1	NA	Left	Temporal	PXA (m)	BRAF V600E(c.1799T>A)	No duplication	3	Present	Recurrence
BT35	MGH	M	24.5	1.9	Right	Temporal	PXA (m)	BRAF V600E(c.1799T>A)	NA	3	Present	Primary
BT36	MGH	F	76.2	5	Right	Parietal	aPXA	No mutations	NA	3	Focal	Primary
BT38	CH	F	18.7	1.2	Right	Frontal	PXA	BRAF V600E(c.1799T>A)	NA	2	Focal	Recurrence
BT39	BWH	M	18.6	2.4	Left	Temporal	PXA (m)	BRAF V600E(c.1799T>A)	No duplication	3	Focal	Primary
BT41	BWH	M	46.6	1.1	Right	Temporal	aPXA	No mutations	No duplication	4	Focal	Recurrence
BT44	BWH	M	86.8	NA	Left	Temporal	aPXA	No mutations	NA	4	Absent	Primary
BT46	CH	F	19.3	3.8	Left	Parietal/Occipital	PXA	No mutations	No duplication	4	Present	Primary
BT47	CH	M	16.5	NA	Right	Temporal	PXA (m)	BRAF V600E(c.1799T>A)	NA	1	Present	Primary
BT48	CH	F	16.3	1	Right	Temporal	PXA	BRAF V600E(c.1799T>A)	No duplication	2	Present	Primary
BT49	BWH	F	30.7	2.1	Left	Temporal	gcGBM arising in PXA	BRAF V600E(c.1799T>A)	NA	4	Focal	Primary
BT52	MGH	F	33.4	4	Right	Temporal	PXA	No mutations	NA	2	Focal	Primary
BT56	BWH	F	62.1	4.6	Right	Frontal	aPXA	No mutations	No duplication	4	Focal	Primary

PXA – pleomorphic xanthoastrocytoma; PXA (m) – pleomorphic xanthoastrocytoma with mesenchymal-like growth pattern; aPXA – anaplastic pleomorphic xanthoastrocytoma; NA – Not assessed.

### SNaPshot Analysis Reveals that BRAF V600E Mutations are Common in PXA

**Figure 2:**
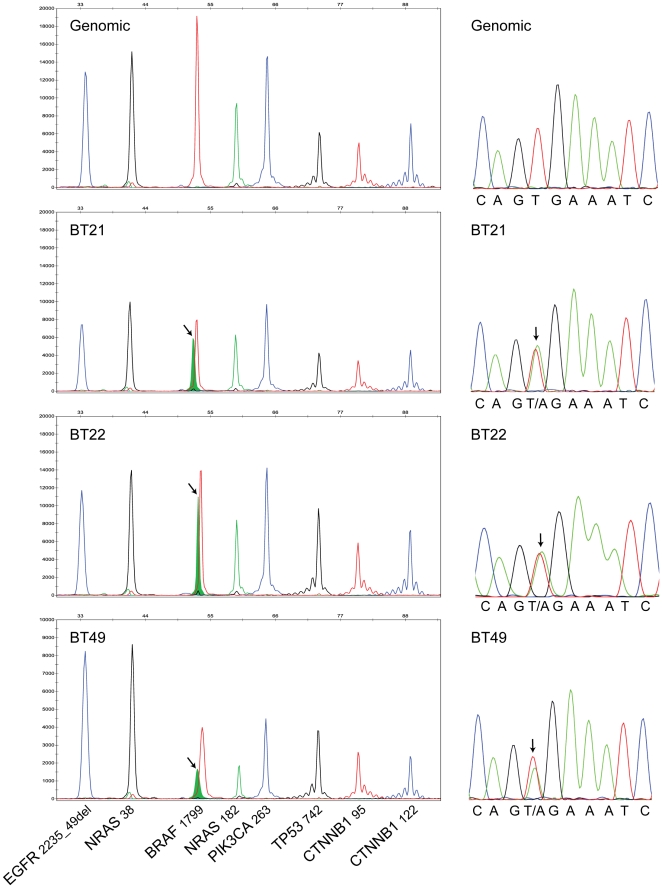
Mutation profiling of pleomorphic xanthoastrocytoma and giant cell glioblastoma arising in a PXA reveals BRAF V600E mutations. The sections on the left illustrate SNaPshot genotyping and the sections on the right depict Sanger sequencing of *BRAF* exon 15 for the same samples. The top panel shows genotyping data obtained with normal male genomic DNA (Promega, Madison, WI). The lower panels illustrate BRAF V600E (c.1799T>A) mutation detection (arrows) in tumor DNA derived from formalin-fixed paraffin-embedded specimens of representative examples of: PXA (BT21), anaplastic PXA (BT22) and gcGBM arising in a PXA (BT49). Assays: (1) *EGFR* 2235_49del R; (2) *NRAS* 38; (3) *BRAF* 1799; (4) *NRAS* 182; (5) *PIK3CA* 263; (6) *TP53* 742; (7) *CTNNB1* 95 and (8) *CTNNB1* 122.

To characterize DNA mutations in this group of PXA tumors we performed SNaPshot multiplexed genotyping (Figure 2). The genotyping analysis queried 60 commonly mutated loci in 15 cancer genes (Table 1) testing for 140 previously described somatic mutations (COSMIC database, v49 release). The BRAF V600E mutation was identified in 12 of the 20 PXA (60%). The average age of patients bearing the BRAF V600E-positive PXA was 26.2 years versus 25.1 years in those without the mutation. The average tumor size at presentation for BRAF V600E-positive tumors was 2.2 cm compared to 2.0 cm for BRAF V600E-negative tumors. In our cohort of 11 males and nine females with a PXA, 73% of males with PXA (8 of 11) had a BRAF V600E mutation while only 44% of females with PXA (4 of 9) had a BRAF V600E mutation. Despite BRAF V600E-positive PXA occurring more frequently in males in our cohort, the association between tumor mutational status and gender was not significant (p value 0.36). Interestingly, the mutation was present in 8 of 9 PXA demonstrating prominent mesenchymal-like growth pattern (PXA (m)), revealing a significant association of BRAF V600E mutation with PXA (m) (p value 0.028). Of the remaining four BRAF V600E-positive PXA cases, two were also present in the temporal lobe, one was present in the frontal lobe and the remaining one originated in the occipital lobe and displayed ganglion cell/neuronal differentiation (BT20). In all, 70% of the PXA originated in the temporal lobe and 83.3% of the BRAF V600E-positive grade II PXAs were located in the temporal lobe. Given the cohort size and the limited availability of follow up data, correlative analysis regarding the prognostic significance of the BRAF V600E mutation was not possible in grade II PXAs. The BRAF V600E mutation was present in only one of the six aPXA, in the single gcGBM arising from a PXA in our cohort and in one of nine additional gcGBM (11%). The aPXA case bearing a BRAF V600E mutation was a recurrence of a grade II PXA and was located in the frontal lobe. The primary tumor for this recurrence was not available for genotyping. The BRAF V600E-positive gcGBM arising in a PXA was also located in the temporal lobe. Neither of these two gcGBM specimens had amplification of the *EGFR* locus.

BRAF V600E mutation has not been previously reported in gcGBM but it has been reported to occur at low frequency in glioblastoma and in one case of gliosarcoma. In total, BRAF V600E mutation in standard glioblastomas has been reported in approximately 1.7% of cases (5 of nearly 300 cases) [25]–[29]. In line with this mutation frequency, review of 62 standard glioblastoma cases that had undergone clinical testing at MGH using SNaPshot mutation profiling identified only one case with a BRAF V600E mutation (1.6%). Of note, SNaPshot clinical testing of these 62 GBMs included the assays depicted in Table 1 except for IDH2. Review of these GBM cases also revealed additional low frequency mutations in cancer genes that have been previously described to be altered in standard GBM (Table 3) including mutations in *IDH1* (3; 4.8%), *KRAS* (1; 1.6%), *PIK3CA* (4; 6.5%), and TP53 (7; 11.3%) supporting the strength of using multiplexed tumor genotyping to identify low frequency mutations, some of which can be treated with targeted therapeutics. Also highlighting the strengths of a multiplexed genotyping approach is the observation that one of the mutations identified in *IDH1* (c.394C>T) results in a R132C substitution that would not be identified by IHC using an IDH1 R132H mutation specific antibody.

**Table 3:** Mutation analysis of GBM.

Gene	Mutation	No. of cases
***BRAF***	p.V600E, c.1799T>A	1
***IDH1***	p.R132C, c.394C>T	1
	p.R132H, c.395G>A	2
***KRAS***	p.G12A, c.35G>C	1
***PIK3CA***	p.R88Q, c.263G>A	3
	p.H1047R, c.3140A>G	1
***TP53***	p.R175H, c.524G>A	1
	p.R248W, c.742 C>T	2
	p.R273C, c.817C>T	2
	p.R273H, c.818G>A	1
	p.R306*(STOP), c.916C>T	1

SNaPshot clinical genotyping of 62 glioblastoma cases from MGH identified cancer gene mutations that confer a favorable prognosis to the patients (IDH1) or that activate pathways targeted by therapeutic agents under clinical development (BRAF, KRAS and PIK3CA).

One TP53 mutation (p.R248Q; c.743G>A) was found in a gcGBM tumor sample but additional mutations profiled by the SNaPshot system were absent in these gcGBM cases. Mutations in the *TP53* gene have been reported in 60–90% of gcGBM cases [30]–[32]. Since the SNaPshot panels used in our experiments cover less than 30% of the *TP53* mutations that have been reported in gcGBM (COSMIC database, v49 release), the odds dictate that we would have detected a *TP53* mutation in about 2 of the 9 cases in this study. Support for the existence of TP53 mutations in many of the gcGBMs analyzed in this study was provided by p53 immunohistochemistry which revealed increased p53 expression in 7 of the 9 (78%) gcGBM.

Interestingly, none of the other mutations in the SNaPshot panel that are commonly found in cancer were present in the PXAs that were analyzed including those described above for standard GBM. Of note, mutations at the most frequently affected amino acid residues in the isocitrate dehydrogenase genes 1 (*IDH1*, R132) and 2 (*IDH2*, R172) and *TP53* (R175, G245, R248, R273 and R306) were not identified in any of PXA samples. In all, this data shows the BRAF V600E mutation is common in PXA but is infrequent in much more aggressive tumors like GBM and gcGBM that enter the differential diagnosis of PXA.

### Phospho-ERK Immunohistochemistry and *BRAF* FISH Analysis

**Figure 3:**
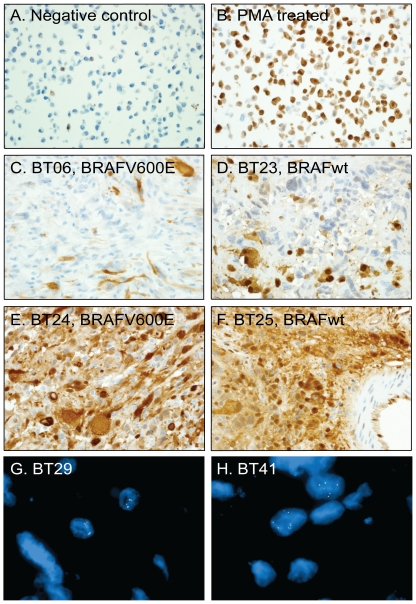
Phospho-ERK (pERK) immunostaining and fluorescence *in situ* hybridization (FISH) for *BRAF*. Validation of the pERK antibody using NIH/3T3 cells that are either untreated (A) or PMA-treated (B). C. pERK IHC of BT06 PXA with a BRAF V600E mutation (IHC semi-quantitative score 1). D. BT23 PXA without BRAF V600E mutation (IHC semi-quantitative score of 2). E. BT24 PXA with a BRAF V600E mutation (IHC semi-quantitative score 4). F. BT25 PXA without BRAF V600E mutation (IHC semi-quantitative score of 4). G. *BRAF* FISH with probes for CEP7 (centromeric control probe - aqua), 5′BRAF (green) and 3′BRAF (red). PXA BT29 demonstrating no duplication of the *BRAF* locus and chromosome 7 disomy. H. PXA BT41, which is the recurrence of BT29, did not have duplication of the *BRAF* locus but demonstrated chromosome 7 polysomy.

To address the functional consequences of BRAF V600E mutations in PXA, we assessed MAPK pathway activation by measuring levels of phospho-p44/42 MAPK (Erk1/2) by IHC. While immunoblot analysis provides a quantitative method for assessing differences in phosphorylation, access to only FFPE and not frozen samples of this uncommon tumor permitted analysis by the less quantitative method of IHC. Staining was scored using a semiquantitive scoring system (0–4 scoring system). Interestingly, the BRAF V600E mutation in grade II PXA did not result in an overt increase in levels of phospho-ERK for mutated tumors (mean score 2.4 for BRAF V600E-positive Grade II PXA and 3.1 for BRAF V600E-negative Grade II PXA; p value 0.13). Representative images of staining are displayed in Figure 3.

Since *BRAF* duplication has been identified as a mechanism for activation of the MAPK pathway in pilocytic astrocytomas [33]–[38] we investigated copy number alterations at the *BRAF* locus by FISH. Results were obtained for seven of the 12 BRAF V600E-positive grade II PXA, for six of the eight BRAF V600E-negative grade II PXA and for three of the five aPXA lacking the BRAF V600E mutation. While some tumors showed polysomy of chromosome 7 (3–5 copies) in a portion of the tumor cells, none of the tumors analyzed by FISH showed *BRAF* duplication (Fig. 3G, H) suggesting that this mechanism does not account for the activation of the MAPK pathway observed in BRAF V600E-negative PXA.

## Discussion

### The BRAF V600E Mutation is Common in PXA

While BRAF V600E mutations have been described as a common mutation in ganglioglioma (GG) by both our group and others [16], [39], the frequency of BRAF V600E mutation in other low-grade gliomas has not been investigated in depth. An individual case with BRAF V600E mutation has been reported for pilocytic astrocytoma [16] and several diffuse astrocytomas (WHO grade II) have also been identified with BRAF V600E mutation [16], [39], [40]. During the preparation of this manuscript, individual cases of WHO grade II PXA with BRAF V600E mutation have been reported with one occurring in the temporal lobe of a 36-year-old woman [39], one in the cerebral cortex of a six-year-old girl [41] and one in the temporal lobe of a 16 year old boy with velocardiofacial syndrome [42]. Notably, we now identify the BRAF V600E mutation in approximately 60% of grade II PXA, a similar mutation rate as seen in malignant melanoma [43]. Also of interest, is that a number of common mutations found in some classes of human glial tumors are absent in our PXA cohort, including mutations in *IDH1* and *IDH2* [24], [44], [45] and that *BRAF* is not duplicated in PXA. Also of note, during the review process of this manuscript, a separate study was published identifying BRAF V600E mutations in 66% of PXA [23], consistent with the findings in our study.

There is variability in the histopathologic presentation of PXA. Common features of PXA include xanthomatous change and marked cellular pleomorphism, however, some tumors demonstrate fascicular or storiform growth of spindle-type cells [1], [7], [46] while others are characterized by a diffuse, glial/fibrillary pattern of growth [1]. Nearly 50% of the tumors in our series displayed a mesenchymal-like growth pattern and were designated PXA (m) (Table 2). All of the PXA (m) tumors originated in the temporal lobe and interestingly, in our series, nearly all (89%) of these PXA (m) tumors harbored BRAF V600E mutations, whereas the mutation was only present in 36% of the remaining PXA. The gcGBM arising in a PXA which harbored a BRAF V600E mutation in our series also arose from a PXA demonstrating a mesenchymal-like growth pattern and was also located in the temporal lobe. Analysis of more PXA cases will be required to see whether the strong association holds between BRAF V600E mutation and PXA (m). Nonetheless, our data seems to support that the classification of PXA, as currently used, is molecularly heterogeneous and may be composed of a group of BRAF V600E-positive PXAs predominantly originating in the temporal lobe that display pleomorphic giant cells, xanthomatous change, and fascicular and storiform growth pattern. This group may be clinically and molecularly distinct from the remaining PXA tumors.

### Diagnostic Considerations

The diagnosis of PXA is traditionally based on examination of H&E-stained sections and reticulin preparations as well as integration of clinical and radiological information [1], [2], [5]. The identification of BRAF V600E mutations as a common mutation in PXA offers an ancillary diagnostic tool that can help discriminate PXA from standard GBM and gcGBM as well as pilocytic astrocytoma. Since a similar percentage of ganglioglioma (GG) harbor BRAF V600E mutations (approximately 60%), the BRAF V600E status will not help to discriminate PXA and GG. While a more complete survey of other uncommon low-grade gliomas is still required, it appears likely that the high frequency of BRAF V600E mutations will be the hallmark of PXA and GG. Interestingly, in addition to the strong clinicopathologic similarities of PXA and GG, composite PXA-GG tumors are also observed, albeit infrequently [47], [48]. One of the cases in our study (BT 20) demonstrates such mixed features and was found to carry a BRAF V600E mutation. This molecular link between PXA and GG further supports the clinicopathologic overlap between these tumors and raises the possibility of a shared molecular pathogenesis/histogenesis.

Additional molecular tests can be used as part of a panel including BRAF V600E analysis in the evaluation of PXA. The absence of *BRAF* duplication in our PXA series can help distinguish PXA from the 40% of non-cerebellar PAs and 80% of cerebellar PAs [37] that have BRAF rearrangements. The absence of *IDH1* and *IDH2* mutations in our PXA cohort suggests that these mutations are rare or not present in PXA, a feature that can help discriminate PXA from the 87.5% of diffuse glioma that bear mutations in these gene [49]. Also, FISH or CISH for *EGFR* can be of assistance since *EGFR* is amplified in 30–40% of GBM [2]. The integrated molecular genetic evaluation of PXA can help facilitate appropriate histologic classification.

### Therapeutic Considerations

The activation of the MAPK pathway in PXA has significant therapeutic implications as well. Moderate to high level expression of phospho-ERK was observed by IHC in the majority of PXA and aPXA suggesting that MAPK signaling is an important component of PXA. These findings suggest that PXA may be responsive to treatment with orally available BRAF kinase inhibitors such as PLX-4032 [50] as well as by HSP90 inhibitors which destabilize mutated BRAF [51], [52]. BRAF V600E-positive melanomas seem to be highly dependent on activation of the MAPK pathway for proliferation and maintenance functions. Preclinical testing in melanoma has demonstrated that PLX-4032 can inhibit the *in vitro* and *in vivo* proliferation of BRAF V600E melanoma cell lines and clinical trials of PLX-4032 have shown clinical responses in BRAF V600E melanoma with the extent of response associated with the level of inhibition of phospho-ERK [50], [53]–[55]. These studies have provided the proof-of-principle for the use of BRAF inhibitors in melanoma, and raise the possibility of using targeted small molecule therapeutics like PLX-4032 in the treatment of PXA and GG, particularly in the setting of recurrence or in cases where noncytotoxic chemotherapy can serve as an adjuvant treatment to permit resection of complex lesions [56]. Most critical now will be to demonstrate sensitivity of BRAF V600E-positive glioma cell lines and tumors to BRAF targeted therapeutics. In addition it will be important to identify and validate other pathways that contribute to the proliferation of PXA and GG. Such studies could provide the rationale for concurrently targeting additional pathways besides MAPK to generate clinical responses and sustained remissions.
